# Long-Term Outcomes of One-Anastomosis Gastric Bypass: A Systematic Review of Studies with at Least 10 Years of Follow-Up

**DOI:** 10.1007/s11695-025-08479-z

**Published:** 2026-01-05

**Authors:** Adam Abu-Abeid, Andrei Keidar, Shiran Gabay, Jonathan Benjamin Yuval, Nir Messer, Mati Shnell, Shai Meron Eldar, Avner Leshem

**Affiliations:** 1 Tel Aviv Sourasky Medical Center, Tel Aviv, Israel; 2 Tel Aviv University, Tel Aviv, Israel

**Keywords:** One anastomosis gastric bypass, Long-term, Weight loss, Type 2 diabetes, Hypertension

## Abstract

One anastomosis gastric bypass (OAGB) is increasingly becoming popular worldwide and is considered a safe and effective procedure. However, data regarding long-term efficacy is lacking. We performed a systematic review of articles reporting outcomes in patients with a minimum follow-up of 10 years. Five retrospective studies comprising 1,750 patients met the inclusion criteria. Overall, the weighted mean percentage of total weight loss was 31.1% ( range 29.6–32.1), and remission rates of type 2 diabetes and hypertension ranged from 70.8 to 90% and 56.7–85%, respectively. Conversion or revisional surgery following OAGB was required in 5.2% ( range 3.3–6.4%) of patients on average. This systematic review suggests that OAGB remains a relatively effective and safe procedure after 10 years.

## Key Points

OAGB resulted in a mean total weight loss of 31.1% in a > 10-year follow-up.

OAGB caused the resolution of obesity related diseases in > 50% of patients.

The rate of conversion surgery was 3.3–6.4%.

OAGB is safe and effective after ten years.

## Introduction

One Anastomosis Gastric Bypass (OAGB) is a hypo-absorptive procedure first described by Robert Rutledge in 1997 and has since evolved into one of the most commonly performed metabolic bariatric procedures worldwide [1, 2]. Its single gastrojejunal anastomosis, shorter operative time, and sustained weight-loss outcomes have contributed to its growing popularity [3, 4].

The prevalence of OAGB continues to rise, and according to the most recent IFSO Global Survey, it now accounts for 7.6% of all bariatric procedures worldwide, reaching 14.9% in Europe and 16% in the Asia-Pacific regions [5].

Long-term outcomes play a key role in assessing the proper safety and efficacy of metabolic and bariatric procedures [6]. OAGB was initially met with skepticism, particularly due to concerns about bile reflux and potential nutritional deficiencies [7, 8]. However, extended follow-up studies are essential to clarify whether these risks translate into significant long-term complications and to determine the durability of weight loss and metabolic benefits. As evidence accumulates from cohorts with more than a decade of follow-up, our understanding of OAGB’s long-term safety profile continues to evolve, helping define its role in the modern era of Metabolic and Bariatric Surgery (MBS).

This systematic review aims to identify and summarize all studies reporting outcomes of OAGB with at least 10 years of follow-up, focusing on weight loss, metabolic outcomes, late complications, nutritional sequelae, and the need for conversion surgery during the follow-up.

## Methods

### Literature Search

A predefined methodological protocol prior to initiating the literature search, including prespecified eligibility criteria, search strategy, outcomes of interest, and planned analytic approach. A systematic search was conducted in PubMed, Embase, and the Cochrane Library using the keywords “long-term, “one-anastomosis gastric bypass, and “mini-gastric bypass.” Only original English-language studies reporting outcomes at 10 years or more after OAGB were included. No date restrictions were applied. The review followed PRISMA guidelines for systematic reviews [9].References of eligible articles were manually screened to identify additional relevant studies. Two independent reviewers performed the title and abstract screening, and discrepancies were resolved by consensus.

### Inclusion and Exclusion Crtieria

Inclusion criteria comprised studies reporting ≥ 10-year outcomes following primary or conversion OAGB in adult patients, using any study design except case reports. Eligible studies were required to provide extractable data on weight-loss outcomes and/or long-term complications. Exclusion criteria included non-English publications, case reports, conference abstracts, reviews, studies with mixed MBS procedures without separate OAGB data, and studies with follow-up shorter than 10 years.

### Data Extraction

Data were extracted using a standardized form and included: first author, year of publication, study design, previous bariatric procedures, preoperative body mass index (BMI), biliopancreatic limb (BPL) length, percentage of total weight loss (%TWL) at 10 years, remission rates of type 2 diabetes (T2D) and hypertension (HTN), complication rates, and the proportion and type of conversion or revisional surgeries.

### Data Analysis

Continuous variables were reported as mean ± standard deviation, and categorical variables as percentages. Weighted means and proportions were calculated according to the sample size of each included study to provide overall estimates.

### Quality Assessment

The methodological quality and risk of bias of the included studies were evaluated using the Newcastle–Ottawa Scale (NOS) for observational studies. Each study was independently assessed by two reviewers, and discrepancies were resolved through discussion and consensus.

## Results

**Fig. 1:**
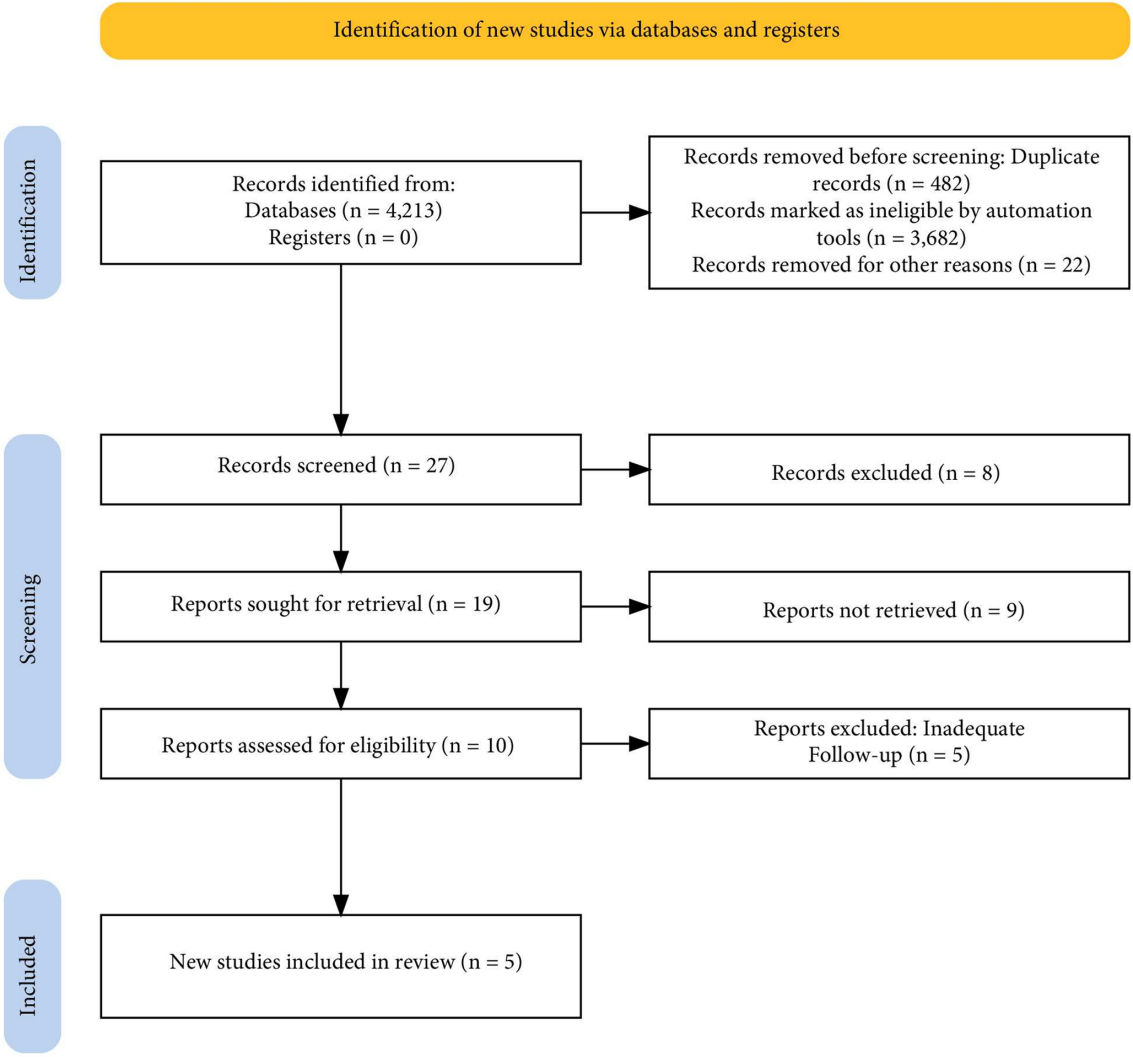
PRISMA flow diagram

Our literature search identified a total of 4,213 articles; 482 duplicates were removed before screening. After removal of case reports, reviews/meta-analyses, and non-English studies, 26 articles on OAGB were screened. The PRISMA flow chart for the study selection is shown in Fig. 1. Five retrospective studies reporting outcomes of OAGB after 10 or more years were included in the present study [10–14].

The sample size of these articles ranged from 63 to 570, comprising 1750 patients in total. Only one paper reported a multicenter experience, while the other four published a single-center case series (Table 1). All articles followed international guidelines on BMI threshold for metabolic surgery, and remission of obesity related diseases was defined according to commonly accepted definitions, with slight variation across articles for T2D and HTN. Remission of OSA and GERD was defined as patient-reported symptoms or medication use.

**Table 1:** Key outcomes of studies reporting ten-year FU after one-anastomosis gastric bypass

Author, year	Number of patients	Study design	Previous bariatric procedure (%)	Mean BMI (kg/m^2^)	Mean BPL length (cm)	Mean TWL (SD)	Last FU BMI (kg/m^2^)	Conversion surgery during FU
Liagre, 2022	405	Retrospective single-center	14.8%	42.5 ± 5.6	150	31.6 ± 11.2	28.8 ± 5.2	26 (6.4%)
Makkapati, 2025	152	Retrospective single-center	0%	48 ± 20	180–200, 250§	32.1 ± 11.5	28.4 ± 6.4	5 (3.3%)
Dowgiałło-Gornowicz, 2025	63	Retrospective multi-center	N/A	39.6 (median)	200	27.6#	N/A	N/A
Carandina, 2021	560	Retrospective single-center	49.6%	44.3 ± 6.7	200	32.1 ± 11.4	31.9 ± 5.2	24 (4.3%)
Allmuhanna, 2021	570*	Retrospective single-center	0%	40.2 ± 11.9	200	29.6 ± 12.5	27.7 5.6	113 (5.1%)

BMI-Body mass index, BPL- Biliopancreatic limb, TWL-Total weight loss, FU-Follow up, N/A -Not available

#median

*Entire cohort was 2223, 570 patients reported to complete ten-year follow-up

§In case common channel was 400 cm, 250 cm BPL was preferred

**Fig. 2:**
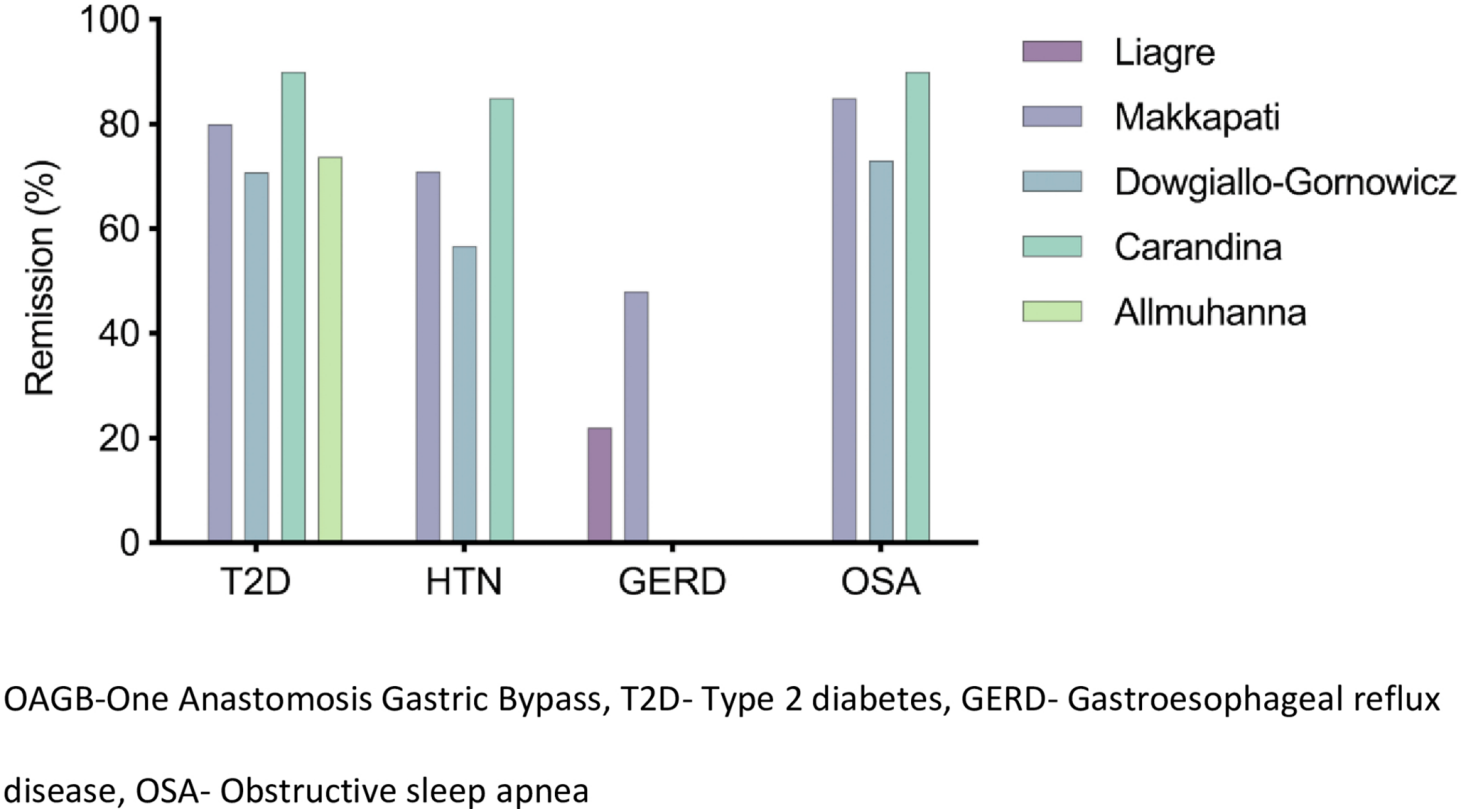
Summary of studies reporting resolution of obesity related diseases in OAGB patients after 10-years follow-up

At 10 years following OAGB, four articles reported mean %TWL ranging from 29.6 ± 12.5 to 32.1 ± 11.5 (Table 1), and the overall weighted mean %TWL was 31.1% [10, 11, 13, 14]. The fifth multicenter study reported a median %TWL of 27.6% in 63 patients [12]. Weighted remission rates of T2D and HTN were 79.4% and 79.9%, respectively. Remission rate of gastroesophageal reflux disease (GERD) and Obstructive sleep apnea (OSA) ranged from 22 to 48% and 73.1–90%, respectively. OAGB was performed as a revisional surgery in 49.6% of patients in one study group (*n* = 560) [13], in 14.8% of patients in another (*n* = 405) [10]. In two other study groups, all OAGB procedures were performed as primary surgery, and one study did not report this variable. Conversion surgery was performed following OAGB in 5.2% (3.3–6.4%) of patients on average. Remission rates of obesity-related diseases are summarized in Fig. 2.

## Discussion

The present systematic review summarizes the available evidence on outcomes of OAGB (both as primary and conversion surgery) after at least ten years of follow-up. Long-term data of this magnitude are rare in the medical literature and provide valuable insight into the durability and safety of OAGB. The studies included consistently demonstrate that the substantial weight loss and metabolic improvements observed in the early years following OAGB can be largely maintained a decade after surgery. At the same time, extended follow-up allows a clearer understanding of the procedure’s potential drawbacks, particularly regarding nutritional deficiencies and bile reflux, which were central to early skepticism about its widespread adoption. Overall, these long-term results contribute to defining the true balance between benefit and risk of OAGB in the modern era of metabolic and bariatric surgery.

### Weight Loss Outcomes

Across the studies analyzed, mean TWL at ten years ranged from approximately 27% to 32%, reflecting a durable and clinically meaningful effect. The rate of conversion surgery was 3–6%. At ten years, OAGB shows good long-term results when compared with other procedures. Vitiello et al. [15] conducted a systematic review on long-term outcomes after Sleeve Gastrectomy (SG) with at least ten-year follow-up; the mean TWL was 24.4% and the need for revisional surgery was nearly 20%. In the SM-BOSS randomized trial, patients were followed up for 10 years, and the mean TWL was 27.7% [16]. In a retrospective cohort analysis of patients undergoing RYGB by Jawhar et al., the average TWL at 10 years was 23.5% and at 15 years was 25.6% [17]. When comparing to single anastomosis duodeno-ileal bypass or duodenal switch, results reported by El Ghazal et al. showed TWL of 38% and 35%, respectively [18]. Overall, these findings suggest that OAGB achieves durable long-term weight loss comparable to, and in some cases slightly exceeding, that of other MBS over a decade of follow-up.

### Remission of Obesity Related Diseases

In the four studies that reported this outcome, comprising 327 patients who had T2D at baseline and who completed a minimum of 10-year follow-up, the T2D remission rate ranged from 70.8% 90%. This rate is comparable to the long-term remission rate of T2D following other bariatric procedures such as RYGB [6, 16] and SG [15], and reflects a slight decrease in the incidence of T2D remission at 5 years following OAGB, mirroring the trend of weight. Of note, heterogeneity in the definition of T2D remission across studies explains some of the differences in T2D remission rates, although those differences are not substantial in our view. Significant long-term remission rates of other obesity-related diseases, such as HTN, GERD, and OSA, further underscore the merits of OAGB.

### Nutritional Outcomes

Although OAGB has shown durable long-term results, concerns about nutritional deficiencies have persisted since its introduction. Excessive biliopancreatic limb length and inadequate postoperative nutritional surveillance have been identified as the main contributors to protein–energy malnutrition and micronutrient deficiencies [19]. Makkapati et al. [11] reported that at 10-year follow-up after OAGB, none of the patients had protein energy malnutrition, iron and calcium deficiency were in 2% of patients, and vitamin D deficiency was prevalent in 15% of patients. Only one patient (0.7%) required reversal of OAGB due to nutritional issues. Almuhanna et al. reported that 51/2223 patients (2.3%) underwent revisional surgery due to malnutrition. Several studies highlighted iron deficiency anemia (IDA) as a significant concern after OAGB. Carandina et al. [13] reported that nearly 3% of patients had severe anemia that required admission with transfusion. Kermansaravi et al. [20] conducted a systematic review of studies reporting iron deficiency (IDA) after OAGB; it was reported that IDA prevalence is 16% and the biliopancreatic limb length predicts IDA rates. We believe that nutritional deficiencies are not uncommon after OAGB, and as with all MBS, patients should be strongly advised to maintain routine follow-up and lifelong vitamin supplementation. The optimal biliopancreatic limb length in OAGB remains a subject of ongoing debate, particularly regarding its impact on nutritional outcomes [21]. Felsenreich et al. [22] reviewed various limb length configurations across gastric bypass procedures and recommended that when constructing a biliopancreatic limb exceeding 200 cm, the total small bowel length should be measured intraoperatively to ensure an adequate common channel is preserved.

### Bile Reflux

Bile reflux is often regarded as one of the main disadvantages of OAGB. Carandina et al. [13] reported that nearly 10% of patients developed bile reflux after 10 years, with a higher incidence in patients after gastric banding. Moreover, 5.7% of patients required conversion to RYGB due to unresponsiveness to medical therapy. In a systematic review by Musella et al. [23] reporting OAGB outcomes with at least 5-year follow-up, the rate of biliary reflux was reported to be 1–10% and was the most common indication for revisional surgery. Bile reflux remains one of the downsides after OAGB and can actually be considered the Achilles Heel. Conservative management of bile reflux typically includes high-dose proton pump inhibitors, sucralfate, and lifestyle modifications such as avoiding meals before bedtime, remaining upright after eating, and elevating the head of the bed during sleep [4]. In case of severe debilitating symptoms, conversion to RYGB is recommended.

### Other Chronic Complications – Marginal Ulcers and Internal Hernia

Marginal ulceration remains a recognized complication following OAGB, with an incidence reported between 1% and 5% after 10 years [11, 13]. Although most cases respond to medical therapy, surgical revision is required in approximately 1–3.5% of patients [13, 14]. The risk appears to be higher among smokers and in those with poor adherence to proton pump inhibitor therapy [24]. Importantly, the reported incidence of marginal ulcers after OAGB does not seem to differ significantly from that observed following RYGB, suggesting that the omega loop configuration itself is not a major independent risk factor [25].

Internal hernia appears to be an uncommon or possibly underreported complication following OAGB. In this systematic review with at least 10 years of follow-up, the incidence ranged between 0% and 3.9%. Liagre et al. [10] compared long-term outcomes of OAGB and RYGB and found that internal hernias were significantly more frequent after RYGB (8.8%) than after OAGB (3.9%; *p* = 0.003). Closure of the mesenteric defect in OAGB is not performed routinely in most studies and remains without a clear consensus among surgeons. Although such closure could theoretically reduce the incidence of internal hernias, there is no clear evidence supporting this.

### Recurrent Weight Gain

The true incidence and management of recurrent weight gain following OAGB are unclear. While long-term data remain limited, recurrent weight gain appears to be relatively uncommon. In the study by Makapati et al., only four patients (2%) were reported to have recurrent weight gain (2%), and 3 underwent conversion surgery, including biliopancreatic limb lengthening and conversion to SADI-S [11]. Similarly, Almuhanna et al. [14] reported a conversion rate of 1% in patients with recurrent weight regain. Interestingly, Liagre et al. demonstrated that revisional surgery for recurrent weight gain was significantly more frequent after RYGB compared to OAGB (2.4% vs. 0.2%) [10]. These findings suggest that OAGB provides durable weight-loss outcomes in the majority of patients.

Despite the encouraging long-term results, several limitations should be acknowledged when interpreting these findings. Most of the available studies are retrospective, single-center series with heterogeneous surgical techniques. In addition, this review is limited by heterogeneity among the included studies, particularly regarding primary versus revisional OAGB and differences in biliopancreatic limb lengths. Most studies did not provide sufficiently detailed or stratified data to allow meaningful subgroup analyses. As a result, the influence of surgical indication and limb length on long-term outcomes remains uncertain. Standardized reporting in future studies is needed to address these important variables. Follow-up completeness varied widely, raising the potential for attrition bias and underreporting of late complications. These methodological differences highlight the need for prospective, multicenter studies with standardized reporting to validate and refine the long-term evidence base for OAGB.

## Conclusion

In conclusion, the available long-term evidence demonstrates that OAGB provides durable weight loss and sustained metabolic improvement, with an acceptable rate of late complications. These findings position OAGB as a safe and effective procedure in the long-term management of severe obesity. Continued prospective studies with extended surveillance are essential to further clarify its long-term nutritional safety and to guide optimization of limb length and postoperative care.

## Data Availability

No datasets were generated or analysed during the current study.
